# The incidence of medium vessel occlusions: a population-based study

**DOI:** 10.3389/fneur.2023.1225066

**Published:** 2023-07-27

**Authors:** Michael Liu, Deena Nasr, Waleed Brinjikji

**Affiliations:** Mayo Clinic, Rochester, MN, United States

**Keywords:** stroke, medium vessel occlusion, epidemiology, vessel occlusion, interventional neurology

## Abstract

**Introduction:**

The incidence of medium vessel occlusion (MeVO) is not well known. The objective of our study is to perform a population-based assessment to estimate the incidence of MeVOs.

**Methods:**

Consecutive patients from Olmsted County, Minnesota who presented for acute ischemic stroke seen at Mayo Clinic Hospital from 1/1/2018 to 12/31/2020 who were found to have a MeVO were included in this study. MeVO was defined as occlusion at or beyond the level of the middle cerebral artery M2 segment, anterior cerebral artery A2 segment, posterior cerebral artery P1 segment, and cerebellar arteries. Census data for Olmsted County was obtained from the United States Census Bureau from the year 2020.

**Results:**

A total of 1,718 patients were screened for the study, 77 patients fulfilled inclusion criteria to be included in the study. Presenting NIHSS was 9 (± 7). The population of Olmsted County was estimated to be 162,847. The incidence rate for MeVO was 16 cases (95% CI 12–19) per 100,000 people per year. Based on estimates of the US population in 2020 of 331,449,281 people, we estimate there are 52,236 (95% CI 40,635-64,002) new cases of MeVOs per year.

**Conclusion:**

As the only stroke center in Olmsted County, we have been able to estimate the incidence of ischemic stroke due to MeVO. While the incidence of MeVOs is less than both large and small vessel occlusions, they still represent a significant proportion of strokes with significant morbidity and mortality that would benefit from further studies in both acute intervention and prevention.

## Introduction

Stroke is a leading cause of morbidity and mortality in the United States affecting more than 795,000 individuals per year. Of all strokes, 87% are ischemic in nature (1). Stroke mechanism is paramount in identifying strategies for acute stroke intervention and long term prevention. While mechanisms such as small vessel and large vessel occlusion (LVO) has been extensively studied, medium vessel occlusion (MeVO) has been less so.

MeVOs are defined as occlusions that are beyond the traditionally defined LVOs and encompass the distal middle cerebral artery (MCA) branches, distal anterior cerebral artery (ACA) branches, posterior cerebral arteries (PCA), and cerebellar arteries. Historically, these occlusions have been thought to be associated with less severe clinical presentations and were not emphasized in the landmark large vessel occlusion thrombectomy trials. Further studies have shown that this is not necessarily the case and MeVOs can be associated with significant morbidity and mortality (2–5). Intravenous thrombolytics have been shown to be beneficial, but only results in recanalization of about one-third to one-half of MeVOs when used without endovascular intervention (4, 6, 7). Endovascular intervention with mechanical thrombectomy was initially more difficult due to the size and often tortuosity of the vessel, increasing the risk of complications. However, as endovascular devices have improved, appropriately selected patients have been shown to have significant benefit with mechanical thrombectomy (2, 6, 8–10).

An integral part of understanding MeVOs includes understanding its epidemiology. Epidemiological data regarding incidence of large vessel and small vessel occlusions is better described. Data regarding the frequency of MeVOs is primarily ascertained from subgroup analysis of larger studies and has been estimated to account for 25–40% of ischemic stroke. It is important to note that these estimates are not population based (6, 11–14). There has been few population-based epidemiological study looking at the frequency of MeVOs to date. The objective of our study is to evaluate the incidence of MeVOs in a population-based study.

## Methods

This is an institutional review board approved retrospective study.

### Participants

Consecutive patients >18 years old who presented to Mayo Clinic Hospital in Rochester, Minnesota with a diagnosis of acute ischemic stroke from 1/1/2018 to 12/31/2020 was screened for this study. Of those patients screened, included participants must have an angiographically proven MeVO and be a resident of Olmsted County at the time of their stroke. MeVO was defined as occlusion at or beyond the level of the following vasculature: MCA M2 segment, ACA A2 segment, PCA P1 segment, and cerebellar arteries. All angiographic images were screened by study personnel. Angiographic techniques include computed tomographic angiography (CTA), magnetic resonance angiography (MRA), and invasive digital subtraction angiography. Demographic and clinical data was obtained from the Mayo Clinic electronic health record.

### Outcomes

The primary outcome measures are epidemiological measures including incidence of MeVO occurrence. Asides from epidemiologic data, clinical and functional outcome measures were captured. Severity of initial presentation as captured by the presenting national institute of health stroke scale (NIHSS) and 90 day modified Rankin Score (mRS) were captured and analyzed as outcome measures.

### Census data

Mayo Clinic is the only stroke center in Olmsted County and captures 100% of acute stroke in the county. Census data for the county as well as the country was obtained from the United States Census Bureau’s most recent data from the year 2020.

### Statistical analysis

Statistical analysis was done with the assistance of SPSS V27 statistical software (IBM Corporation, Armonk, New York). Comparison between two groups were completed using two-sample T-tests for continuous variables and chi-square analysis for categorical variables. Logistic regression modeling the effect of various clinical factors and clinical outcomes was also performed. Census data for Olmsted County as well as the United States was used to determine epidemiologic results of interest for this study.

## Results

**Figure 1:**
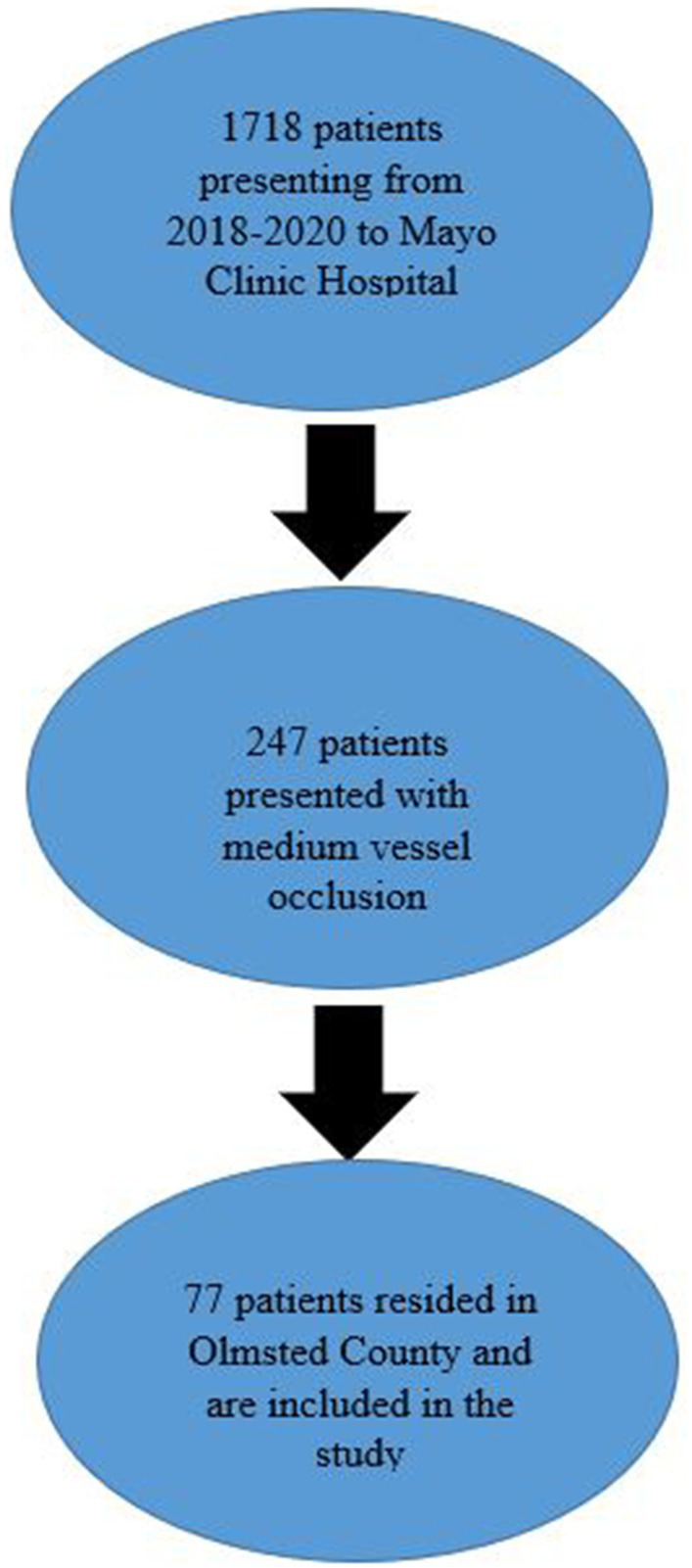
Patient selection flowsheet.

A total of 1,718 patients presented to Mayo Clinic Hospital from 1/1/2018 to 12/31/2020. A total of 247 patients presented with MeVOs. Of those patients, 77 lived in Olmsted County at the time of their stroke and were included in the study (Figure 1). Of those patients, the average age was 74 years old (± 14 years), 44 (57%) were female, 67 (87%) were Caucasian, and 55% were former or active smokers. 87% of the patients had a diagnosis of hypertension, 90% had a diagnosis of hyperlipidemia, and 36% had a diagnosis of diabetes. The average presenting NIHSS was 9 (± 7) (Table 1).

**Table 1:** Baseline characteristics of the study population.

Baseline characteristics	Patient cohort (*N* = 77)
Age, mean (SD)	74 (14)
Female, n (%)	44 (57)
Caucasian, n (%)	67 (87)
Smoking	
Active, n (%)	13 (17)
Former, n (%)	29 (38)
Hypertension, n (%)	67 (87)
Hyperlipidemia, n (%)	69 (90)
Diabetes, n (%)	28 (36)
Presenting NIHSS, mean (SD)	9 (7)
Anterior circulation, n (%)	50 (65)
Laterality: right, left, bilateral, n (%)	39 (51), 37 (48), 1 (1)
Proximal MeVO (M2 or P1), n (%)	41 (53)

Of 77 patients, 36 patients (47%) had an occlusion at the M2 segment of the MCA, 5 (7%) had an occlusion of the P1 segment of the PCA. 1 (1%) patient had bilateral occlusions and 39 (51%) patients had right sided occlusions. Fifty patients (65%) had anterior circulation occlusions. Please see Table 2 for further details regarding the vessel occlusion territories. When comparing groups and their presenting mean NIHSS there was no significant relationship between anterior or posterior circulation (*p* = 0.684), left sided or right sided occlusion (*p* = 0.600), or more proximal occlusions (P1 and M2 territories) (*p* = 0.668). 12 (16%) patients received thrombolysis and 6 (8%) patients received mechanical thrombectomy. Of those who received acute therapies, 3 (4%) patients received both thrombolysis and mechanical thrombectomy.

**Table 2:** Medium vessel occlusion territories.

Left MCA M2, n (%)	19 (25)
Left MCA M3, n (%)	6 (8)
Left PCA P1, n (%)	4 (5)
Left PCA P2, n (%)	3 (4)
Left PCA P3, n (%)	3 (4)
Left PICA, n (%)	2 (3)
Right ACA A2, n (%)	2 (3)
Right AICA, n (%)	3 (4)
Right MCA M2, n (%)	17 (22)
Right MCA M3, n (%)	4 (5)
Right MCA M3, Left ACA A3, n (%)	1 (1)
Right MCA M4, n (%)	1 (1)
Right PCA P1, n (%)	1 (1)
Right PCA P2, n (%)	5 (7)
Right PCA P4, n (%)	1 (1)
Right PICA, n (%)	5 (7)

ACA, Anterior Cerebral Artery; AICA, Anterior Inferior Cerebellar Artery; MCA, Middle Cerebral Artery; PCA, Posterior Cerebral Artery; PICA, Posterior Inferior Cerebellar Artery.

90-day mRS was captured in only 35 patients with a median mRS of 3 (IQR 1–6). 11 (14%) of patients had died. Good functional status as determined by mRS 0–2 was seen in 14 (40%) of individuals. By logistic regression, elevated admission NIHSS (OR 1.18, CI 95% 1.04–1.345, *p* = 0.01) was significantly associated with poor functional outcome. Laterality (OR 0.75, CI 95% 0.19–2.96 *p* = 0.682), anterior vs. posterior (OR 0.533, CI 95% 0.13–2.21, *p* = 0.386), more proximal occlusions (P1 and M2 territories) (OR 0.563, CI 95% 0.14–2.21, *p* = 0.409), thrombolysis (OR 0.563, CI 95% 0.13–2.48, *p* = 0.447), and thrombectomy (OR 1.00, CI 95% 0.15–6.91, *p* = 1.00) were all not associated with good functional status.

The 2020 United States Census data showed that the population of Olmsted County was 162,847 individuals. With the 77 MeVOs captured over 3 years, the incidence was calculated to be 16 cases (95% CI 12–19) per 100,000 people per year. The incidence of anterior circulation MeVOs was calculated to be 10 cases (95% CI 7–13) per 100,000 people per year and for posterior circulation MeVOs, 6 cases (95% CI 3–8) per 100,000 people per year. Using census data, the population of the United States has been estimated 331,449,281 individuals. Extrapolating our data yields an estimated of 52,236 (95% CI 40,635-64,002) MeVOs in the United States per year (Table 3).

**Table 3:** Incidence of medium vessel occlusion.

3-year cumulative acute ischemic strokes presenting to Mayo Clinic Hospital, n	1,718
3-year cumulative medium vessel occlusions presenting to Mayo Clinic Hospital, n	247
3-year cumulative medium vessel occlusions presenting to Mayo Clinic Hospital who live in Olmsted County, n	77
Incidence of medium vessel occlusions, cases per 100,000 per year (SD)	16 (12–19)
Incidence of anterior distribution medium vessel occlusions, cases per 100,000 per year (SD)	10 (7–13)
Incidence of posterior distribution medium vessel occlusions, cases per 100,000 per year (SD)	6 (3–8)
Estimated number of medium vessel occlusions in the United States per year	52,236 (40,635–64,002)

## Discussion

Our study was aimed at understanding the epidemiology of MeVOs. Mayo Clinic in Rochester, Minnesota is in the unique position of being the only stroke center in Olmsted County, and we have been able to leverage these circumstances to provide a population estimate regarding the incidence of MeVOs. The rate of MeVOs has been found to be 16 cases (95% CI 12–19) per 100,000 people per year leading to 52,236 cases (95% CI 40,635–64,002) nationally per year. This is considerably greater than other estimates of only M2 occlusion which has been estimated to occur at a rate of 7 cases (95% CI 5–9) per 100,000 people per year (12). Our data is overall similar to recently published epidemiologic data from a different population which estimated the incidence of MeVOs to be 20 (95% CI 14–29) (15). The incidence is still considerably lower than large vessel occlusion which has been estimated at a rate of 24 cases (95% CI 20–28) per 100,000 people per year and also lacunar stroke which has wide estimates between 33 cases (95% CI 30–36) and 52 cases (95% CI 36–68) (16–18).

Patients suffering from MeVOs in this cohort showed significant presenting symptoms with an average NIHSS of 9 (± 7). Assessing for significant differences based on location of the occlusion did not show significant variation of presenting severity. Significant morbidity and mortality were also noted with a median mRS of 3 (IQR 1–6) and a 14% mortality rate in this cohort. This severity is in accordance with the literature that has reported (10). The severity coupled with the relatively high incidence rate makes it clear that MeVOs play a significant role in stroke morbidity and mortality and more research is needed in terms of management in both acute and preventative phases.

Limitations of this study includes the generalizability of the study. Olmsted County is predominantly Caucasian as reflected in our data set and does not account for the often-higher rates of stroke that has been documented in other ethnic groups. Median household income was also higher in Olmsted County ($80,403 vs. $67,521) which may translate to better access to healthcare. Geographically, Olmsted county is not located in the “stroke belt” which historically has seen higher rates of stroke. As a result, as we extrapolate our regional data to national data, our findings may underestimate the true incidence of MeVOs. Not all patients received intracranial angiographic imaging at the time of admission due to expert opinion and often times in favor of other imaging modalities such as a carotid ultrasound. This included 312 (18%) of our cohort and would again also possibly lead to an underestimate of the true incidence in our study. Population based estimates from other centers would yield a more diverse population and when pooled would yield a more accurate estimate. Outcome measures were not the primary focus of the study and is limited by the small cohort and retrospective nature of this study. The variability in definition of MeVO among the literature also presents a limitation.

## Conclusion

As the only stroke center in Olmsted County, we are able to provide a population-based estimate of the incidence rate of MeVOs. While the incidence is less than large and small vessel occlusions, we conclude that MeVOs still play a large role as a stroke mechanism both in terms of their incidence and severity. Further studies are required to understand ideal management for these patients.

## Data availability statement

The raw data supporting the conclusions of this article will be made available by the authors, without undue reservation.

## Ethics statement

The studies involving human participants were reviewed and approved by the Mayo Clinic Institutional Review Board. Written informed consent for participation was not required for this study in accordance with the national legislation and the institutional requirements.

## Author contributions

ML and WB conducted data abstraction. All authors contributed to hypothesis generation and manuscript preparation.

## Conflict of interest

The authors declare that the research was conducted in the absence of any commercial or financial relationships that could be construed as a potential conflict of interest.

## Publisher’s note

All claims expressed in this article are solely those of the authors and do not necessarily represent those of their affiliated organizations, or those of the publisher, the editors and the reviewers. Any product that may be evaluated in this article, or claim that may be made by its manufacturer, is not guaranteed or endorsed by the publisher.
